# Characterization of *Plasmodium falciparum* Adenylyl Cyclase-β and Its Role in Erythrocytic Stage Parasites

**DOI:** 10.1371/journal.pone.0039769

**Published:** 2012-06-26

**Authors:** Eric Salazar, Erin M. Bank, Nicole Ramsey, Kenneth C. Hess, Kirk W. Deitsch, Lonny R. Levin, Jochen Buck

**Affiliations:** 1 Tri-Institutional MD-PhD Program, Weill Medical College and Graduate School of Medical Sciences of Cornell University, New York, New York, United States of America; 2 Department of Pharmacology, Weill Medical College and Graduate School of Medical Sciences of Cornell University, New York, New York, United States of America; 3 Department of Microbiology and Immunology, Weill Medical College and Graduate School of Medical Sciences of Cornell University, New York, New York, United States of America; University of Melbourne, Australia

## Abstract

The most severe form of human malaria is caused by the parasite *Plasmodium falciparum*. The second messenger cAMP has been shown to be important for the parasite’s ability to infect the host’s liver, but its role during parasite growth inside erythrocytes, the stage responsible for symptomatic malaria, is less clear. The *P. falciparum* genome encodes two adenylyl cyclases, the enzymes that synthesize cAMP, *Pf*ACα and *Pf*ACβ. We now show that one of these, *Pf*ACβ, plays an important role during the erythrocytic stage of the *P. falciparum* life cycle. Biochemical characterization of *Pf*ACβ revealed a marked pH dependence, and sensitivity to a number of small molecule inhibitors. These inhibitors kill parasites growing inside red blood cells. One particular inhibitor is selective for *Pf*ACβ relative to its human ortholog, soluble adenylyl cyclase (sAC); thus, *Pf*ACβ represents a potential target for development of safe and effective antimalarial therapeutics.

## Introduction

Malaria remains a major burden in the developing world, causing approximately 1 million deaths per year. It is a vector-borne disease caused by protozoan parasites of the genus *Plasmodium*, the most lethal of which is *Plasmodium falciparum.* A diverse array of protozoal, fungal, and bacterial pathogens, including *Plasmodium spp.,* depend upon the ubiquitous second messenger cyclic adenosine monophosphate (cAMP) for survival and environmental sensing [1]. In fact, two stages of the *Plasmodium* life cycle appear to depend upon cAMP: Sporozoites require cAMP generation for host cell invasion [2], and previous reports suggest that cAMP effectors play an important role in the asexual red blood cell stage of the life cycle. Specifically, inhibition of cAMP-catabolizing phosphodiesterases (PDEs) or addition of membrane-permeable cAMP analogs increase the percentage of schizonts in asynchronous, erythrocytic cultures of *P. falciparum* [3], and treatment of erythrocytic stage cultures with either pharmacological or genetic inhibitors of the main effector of cAMP, Protein Kinase A (PKA), inhibit growth [4], [5]. While these data reveal that the cAMP pathway is required for progression through the erythrocytic, asexual stage of the life cycle, the stage of the life cycle that causes symptomatic malaria, it remains unclear how cAMP levels are controlled during this period.

cAMP is synthesized by adenylyl cyclases (AC), and the *P. falciparum* genome encodes two such enzymes, *Pf*ACα and *Pf*ACβ. Both enzymes contain class IIIB catalytic domains similar to mammalian soluble adenylyl cyclase (sAC) [6]. Mammalian sAC is structurally, molecularly, and biochemically distinct from other mammalian adenylyl cyclases, which are transmembrane proteins regulated by heterotrimeric G proteins (tmACs). Unlike tmACs, mammalian sAC is directly regulated by bicarbonate. In physiological systems, bicarbonate is in nearly instantaneous equilibrium with CO_2_ and intracellular pH (pHi) due to the action of carbonic anhydrases [7]; thus, mammalian sAC serves as a physiological CO_2_/HCO_3_^−^/pHi sensor [8], [9], with specific roles in sperm activation [10], [11], ciliary beat frequency in bronchii [12], pH homeostasis in epididymis [13], kidney [14], [15], and shark gill [16], metabolism [17], and aqueous humor formation in the eye [18].

*Pf*ACα and *Pf*ACβ differ in their modular architecture. *Pf*ACα. contains six predicted transmembrane domains and a single carboxy-terminal catalytic domain homologous to sAC-like ACs. The motifs required for metal cofactor binding, substrate binding, and catalysis are contained within this single catalytic domain, suggesting that this enzyme functions as a homodimer [19]. In contrast, *Pf*ACβ has no predicted transmembrane regions and possesses two sAC-like AC catalytic domains. *Pf*ACβ and ACβ orthologs from other *Plasmodium spp.* possess all the motifs required for catalytic activity, but they are spread across the two presumptive catalytic domains suggesting that catalysis requires intramolecular heterodimerization, similar to mammalian sAC [20]. In addition, these ACs possess a threonine residue which is thought to be predictive for bicarbonate regulation in sAC-like ACs [21]. Unlike other adenylyl cyclases including ACβ orthologs from other *Plasmodium spp*., each catalytic domain of *Pf*ACβ is interrupted by blocks of highly charged stretches of amino acids, which are encoded by low complexity regions of unknown function prevalent throughout the *P. falciparum* genome.

*Pf*ACα has been studied both *in vivo* and *in vitro*. *Pf*ACα is a predicted bifunctional protein comprising both a K^+^ channel and an AC that is conserved in alveolata protozoans [22]. *Pf*ACα transcripts are abundant in sexual stage gametocytes [19], suggesting a possible role during sexual stages. Additionally, ACα proteins in *Plasmodium spp.* appear to play a role during the liver sporozoite stage. Specifically, *P. berghei* sporozoites deficient in ACα were shown to have reduced infectivity of cultured hepatocytes and reduced liver infectivity in a mouse model, but they were viable and exhibited normal growth during asexual, erythrocytic growth [2]. In contrast, *Pf*ACβ has not yet been heterologously expressed or biochemically characterized, and attempts to generate *Pf*ACβ-deficient parasites using protocols that demand growth of the haploid mutant parasite in erythrocyte cultures were repeatedly unsuccessful [2]. Interestingly, its mRNA is highly expressed during the erythrocyte stage; *Pf*ACβ transcript levels begin to increase in the trophozoite stage and peak during schizogeny [23], [24].

We took advantage of a number of small molecule inhibitors of sAC-like adenylyl cyclases to identify the essential source of cAMP during erythrocytic growth. Three distinct AC inhibitors blocked growth of *P. falciparum* inside red blood cells. We established conditions for *in vitro* characterization of *Pf*ACβ. and we tested sensitivity of these three inhibitors against the *in vitro* AC activities of both *Pf*ACα and *Pf*ACβ. Consistent with the differential expression patterns of the two cyclases, only *Pf*ACβ proved to be sensitive to all three, providing strong evidence that it is the source of cAMP essential during erythrocytic growth. Interestingly, one of the three inhibitors was also selective for *Pf*ACβ relative to mammalian sAC demonstrating that small molecules can distinguish between the parasite and host enzymes. These data define *Pf*ACβ as a target for development of novel antimalarial therapeutics.

## Results and Discussion

**Figure 1:**
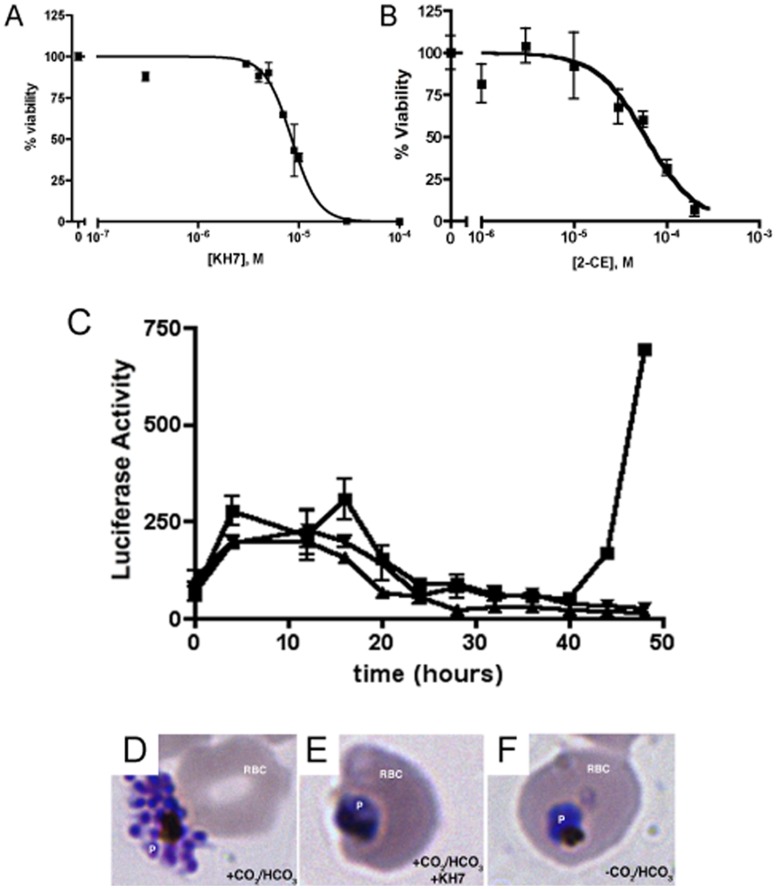
Adenylyl cyclase inhibitors decrease parasite viability. (A) KH7 and (B) 2-CE decrease parasite viability in culture. Reactions were performed in triplicate. Best-fit curves were generated by Prism; error bars represent s.e.m. (C) Luciferase expression in synchronized parasites maintained under normal culture conditions (▪), in the presence of 10 µM KH7 (_←_), or in the absence of supplemental CO_2_/HCO_3_^−^ (_↔_). Samples were collected in triplicate. Luciferase activity is elevated between 4–16 hours due to increased promoter activity during primary round of infection. The peak of luciferase activity seen at ∼44 hr under normal culture conditions, but absent in the absence of CO_2_/HCO_3_^−^ or presence of KH7, reflects reinvasion into RBCs. The graph was prepared with Prism software; error bars represent s.e.m of triplicate wells in the representative experiment. (D) Microscopic evaluation of Giemsa-stained parasites at 44 hr reveals parasites (P) maintained in normal culture completed mitosis and newly released merozoites are poised to reinvade new RBCs. Parasites treated with KH7 (E) or grown in low CO_2_/HCO_3_^−^ conditions (F) never form schizonts.

We have identified two, structurally distinct inhibitors of sAC-like ACs; catechol derivatives of estrogen and KH7 (Figure S1). Catechol estrogens (CEs), such as 2-hydroxyestradiol (2-CE), inhibit Class III ACs, including mammalian and bacterial sAC-like ACs, by chelating the catalytic magnesium ion in the active site [25]. The second structurally unrelated inhibitor, KH7, was identified as a potent, specific inhibitor of mammalian sAC [11], [26], [27] in a small molecule screen [11] and was subsequently found to inhibit a number of bicarbonate-sensitive ACs [16], [28]. To determine the effect of these compounds on parasite growth and viability inside red blood cells, we measured the luminescence of the wild-type NF54 *P. falciparum* strain transfected with the pHLIDH plasmid, which constitutively expresses firefly luciferase [29]. The luminescence of this parasite strain directly corresponds to the measures of viability determined with the widely-used tritiated hypoxanthine-uptake assay [30] (Figure S2). Both KH7 and 2-CE killed rapidly (Figure 1A,B) [LD_50_ = 8.5 µM (95% C.I. = 7.8–9.2 µM) for KH7 and 60 µM (95% C.I. = 43–90 µM) for 2CE] with death observed within a single replicative cycle (48 hours) of synchronized parasites (Figure 1C). Giemsa-stained slides prepared from parasites treated with KH7 revealed condensed, pyknotic parasites (Figure 1E), confirming that these compounds lead to rapid parasite death rather than simply inhibiting proliferation or reporter activity.

As a reference, the terminal phenotype of KH7-killed parasites was indistinguishable from that of parasites maintained in the absence of CO_2_/HCO_3_^−^. Synchronized cultures grown in CO_2_/HCO_3_^−^ in the presence of the inhibitor KH7 or grown in the absence of CO_2_/HCO_3_^−^ lacked the burst of luciferase due to the reinvasion observed in normal cultures (Figure 1C,D). Microscopic evaluation confirmed that the drug-treated parasites (Figure 1E) resembled dead CO_2_/HCO_3_^–^ depleted parasites (Figure 1F); neither formed merozoites, indicating they had not completed schizogeny. In addition, we tested KH7 against a chloroquine-resistant *P. falciparum* strain (Dd2), and it was lethal, as determined microscopically, with similar efficacy as observed against the chloroquine-sensitive NF54 strain (data not shown).

**Figure 2:**
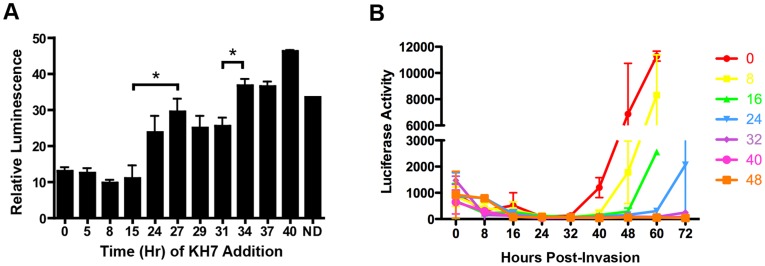
*PfAC* activity is required in early-mid erythrocytic stages. Separate cultures of 1% parasitemia were split from a single synchronized culture. (A) 100 µM KH7 was added to individual cultures at the times indicated. Luminescence was read in duplicate samples from each culture taken after 52 hours; “relative luminescence” reflects luminescence readings relative to luminescence in wildtype C3/NF54 parasites. *, p<0.05 unpaired, two-tailed t-test. (B) Synchronized parasite cultures were maintained in the presence of 100 µM KH7 (orange square); drug was removed at 0 hrs (red circle), 8 hrs (yellow square), 16 hrs (green triangle), 24 hrs (blue triangle), 32 hrs (purple diamond); 40 hrs (pink circle) or 48 hrs (orange square). Luminescence was measured at the times indicated on the x-axis. Graphs were made with Prism software. Error bars represent s.e.m. of duplicate samples from the representative experiment.

In order to determine the temporal effect of KH7 on synchronized parasites, we added KH7 to synchronized cultures at different time points throughout the cell cycle (Figure 2A). Addition of KH7 in the first 24 hours of the cell cycle led to complete cell cycle arrest. However, if KH7 was added to the culture at a point well into schizogeny (34 hours), parasites were able to complete the cell cycle and invade new erythrocytes. In a complementary experiment to determine a “window of KH7-sensitivity,” synchronized cultures were incubated in the presence of KH7 for various times, at which point the drug was washed out and cultures were grown for the remainder of a 48-hour cell cycle. When KH7 was removed at 24 hours or before, cultures were able to progress through the cell cycle, reinvade erythrocytes, and enter G1 (Figure 2B). If KH7 remained on cultures past 24 hours, parasites appeared unable to recover within the 48-hour culture period. These data demonstrate that parasites are most sensitive to KH7 at 24–31 hours post-invasion. This corresponds to the period in the cell cycle during which *Pf*ACβ mRNA levels are beginning to rise dramatically (Figure S3).

**Figure 3:**
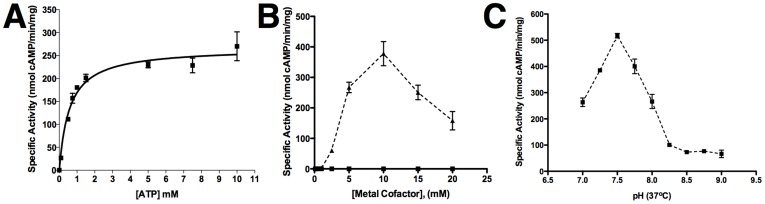
*In vitro* adenylyl cyclase activity of GST-*Pf*ACβ_1-785_. (A) Adenylyl cyclase activity of purified GST-*Pf*ACβ_1-785_ was assessed with increasing concentrations of substrate ATP. Mn^2+^ was kept constant at 20 mM. The Michaelis constant was determined to be 0.57 mM (95% CI = 0.36 mM to 0.8 mM). Vmax was 266.7 nmol cAMP/min/mg (95% CI = 241.9 to 291.6). (B) Adenylyl cyclase activity was assessed over a range of Mn^2+^ (triangles; dotted line) and Mg^2+^ (squares; solid line) concentrations from 0.1 mM to 20 mM. ATP concentration was kept constant at 2.5 mM. Activity was only detectable with Mn^2+^ as a cofactor, and optimal Mn^2+^ was 10 mM providing a ratio of Mn^2+^:ATP = 4∶1. (C) pH optimum of GST-*Pf*ACβ_1-785_. Adenylyl cyclase assays were conducted over a pH range from 7 to 9 with 50 mM Tris buffer. A sharp pH optimum is evident at pH = 7.5. A shift in pH of 0.5 units resulted in a reduction of reaction velocity by ∼½.

We next sought to determine whether the *in vitro* activities of *Pf*ACα and/or *Pf*ACβ were sensitive to 2-CE and KH7. *Pf*ACα has been heterologously expressed and characterized previously [22], but the *in vitro* activity of *Pf*ACβ has not yet been demonstrated. We expressed a synthetic gene encoding the catalytic domains of *Pf*ACβ. AA 1–785) with mammalian codon usage as a fusion protein with a carboxy-terminal glutathione-S-transferase (GST) using a baculovirus (BV) expression system. GST-*Pf*ACβ_1-785_ was soluble, and we were able to purify it only under high salt conditions (Figure S4). This high salt requirement for GST-*Pf*ACβ_1-785_ solubility may be due to the blocks of charged amino acids inserted into its catalytic domains. Similar to other sAC-like ACs [21], [31], [32], [33], [34], including *Pf*ACα [22], which exhibit much greater activity using Mn^2+^-ATP as a substrate relative to Mg^2+^-ATP, purified GST-*Pf*ACβ_1-785_ was active in the presence of Mn^2+^-ATP (Figure 3A). We were unable to detect measurable activity in the presence of Mg^2+^-ATP (Figure 3B). A similar Mn^2+^-ATP-dependency was observed in assays of AC activity in erythrocytic stage *P. falciparum* lysates [35].

GST-*Pf*ACβ_1-785_ displayed Michaelis-Menten kinetics with a lack of cooperative binding of substrate at the active site (Figure 3A). The enzyme has an apparent Michaelis constant (Km) for substrate ATP of ∼0.6 mM using Mn^2+^ as a cofactor with a maximum reaction velocity of ∼265 nmol cAMP/min/mg. This Km value is similar to that obtained for human sAC (0.9 mM) [34]. The optimal ratio of divalent cation (Mn^2+^) to substrate (ATP) was 4∶1 (Figure 3B), similar to mammalian sAC [34], and GST-*Pf*ACβ_1-785_ displayed minimal ability to produce cGMP when supplied with GTP as substrate (data not shown). Mammalian sAC is directly regulated by bicarbonate [34], [36] and calcium [34], [37], and the threonine residue thought to be predictive of bicarbonate stimulation [21] is found in *Pf*ACβ and ACβ orthologs from other *Plasmodium spp*. However, because bicarbonate precipitates in the presence of Mn^2+^, and because we found bicarbonate and calcium activation to be unique to Mg^2+^-ATP-dependent activity in mammalian sAC, we were unable to explore bicarbonate- or calcium-responsiveness of BV-expressed GST-*Pf*ACβ_1-785_. Instead, we explored the pH responsiveness of GST-*Pf*ACβ_1-785_.

In contrast to mammalian sAC, GST-*Pf*ACβ_1-785_ exhibited a strong pH dependence (Figure 3B) [36]. Varying the reaction pH from 7 through 9 revealed a pH optimum of 7.5, and activity decreased sharply at both higher and lower pH values. Thus, *Pf*ACβ activity will be sensitive to changes in pHi, which, in physiological systems, is dependent upon the carbonic anhydrase-mediated equilibrium between CO_2_, bicarbonate, and protons. It is important to note that the pH dependence observed for *Pf*ACβis strikingly similar to the pH dependence of *P. falciparum* in culture. When pH of growth media is maintained between 7.1 and 7.5, parasitemias increase 20–30 fold after three days, with sharp reductions in yield outside of this pH range [38]. During the trophozoite stage, when *Pf*ACβ mRNA is first expressed [23], [24] (Figure S4), the intracellular pH (pHi) of parasites is approximately 7.3 [39], [40], [41]. Therefore, we speculate that *Pf*ACβ functions as the parasite’s pH sensor during growth inside red blood cells.

**Figure 4:**
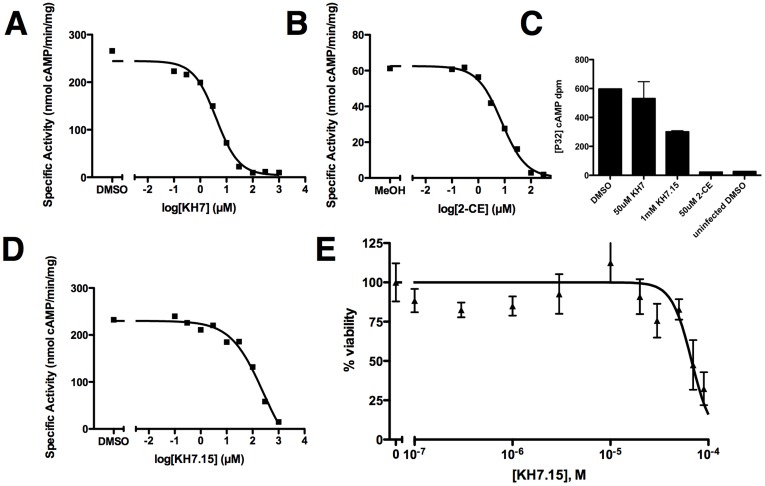
Inhibition of GST-*Pf*ACβ_1-785_ by KH7, KH7.15, and 2-CE. *Pf*ACβ activity was assayed in the presence of increasing concentrations of (A) KH7, (B) 2-hydroxyestradiol (2-CE), or (D) KH7.15. Vehicle control is indicated as the untreated value. Approximate IC_50_s for KH7, 2-CE, and KH7.15 were 5 µM, 8 µM, and 150 µM, respectively. The level of untreated activity was lower in methanol (2-CE vehicle)-treated samples. (C) Activity in *Pf*ACα-expressing Hi5 cells was assayed in the presence of 50 µM KH7, 1 mM KH7.15, and 50 µM 2-CE. Also shown is activity in uninfected Hi5 cells. Values represent averages (with standard deviations indicated) of four independent determinations of cAMP accumulated over 20 minutes. (E) KH7.15 decreases parasite viability in culture (LD50 = 67 µM, 95% C.I. = 58–78 µM).

GST-*Pf*ACβ_1-785_ activity was inhibited by both KH7 and 2-CE with affinities that reflect their observed efficacies in culture. KH7 inhibited GST-*Pf*ACβ_1-785_ with an IC_50_ of 5 µM, and 2-CE showed inhibition with an IC_50_ of 8 µM (Figure 4A,B). In contrast, although *Pf*ACα adenylyl cyclase activity was inhibited by 2-CE, it was largely insensitive to KH7 (Figure 4C). Thus, among adenylyl cyclases in *P. falciparum,* only *Pf*ACβ is inhibited by the two structurally unrelated inhibitors which kill parasites in erythrocytic cultures.

While these data suggest *Pf*ACβ may be a relevant target for killing malaria parasites inside red blood cells, both KH7 and 2-CE are also known to inhibit mammalian sAC, leaving open the possibility that host red blood cell sAC may be the relevant target of these compounds. To address this concern, we sought to identify a PfACβ selective inhibitor. During our screen to identify KH7 as a mammalian sAC inhibitor, we tested numerous KH7-like compounds (Figure S1). Most of the KH7-like compounds were ineffective against sAC-like cyclases, and these proved to have little effect on *P. falciparum* growth (Figure S5). However, one KH7-like compound, KH7.15, which is inert against mammalian sAC [27], inhibited GST-*Pf*ACβ with an IC_50_ of 150 µM (Figure 4D). KH7.15 killed parasites (Figure 4E) with a similar efficacy [LD50 = 67 µM (95% C.I. 58–78 µM)] as it inhibited *Pf*ACβactivity *in vitro*. The fact that parasites were killed by two structurally unrelated inhibitors (2-CE and KH7) and by a third inhibitor (KH7.15) selective for *Pf*ACβ relative to both *Pf*ACα and to the host sAC suggest that *Pf*ACβ is the relevant target of these compounds and is essential for parasite growth inside red blood cells.

Our data include the first characterization of *Pf*ACβ and suggest that *Pf*ACβ is essential for erythrocytic-stage parasite viability. We have demonstrated *Pf*ACβ is biochemically distinct from other Class IIIb adenylyl cyclases and exhibits significant pH-sensitivity. Additionally, we have shown that small molecule inhibitors can distinguish *Pf*ACβ from mammalian sAC. Although the profile of KH7.15 is not ideal for clinical use, the data presented here provide proof-of-principle that *Pf*ACβ can be selectively targeted, thereby identifying it as a therapeutic target for a new class of anti-malarial drugs.

Although effective pharmacological therapies for malaria exist, the widespread and expanding resistance to these drugs demands new approaches to therapeutic intervention. The spread of multi-drug resistant strains of *P. falciparum* threatens to increase the malaria burden, and novel therapeutics to combat malaria are desperately needed. This work is an initial step in attempts to address that need by defining *Pf*ACβas a novel, attractive therapeutic target.

## Materials and Methods

### Compounds

KH7 and KH7.15 were synthesized by the Milstein Chemical Core Facility of Weill Medical College of Cornell University, and other KH7-like compounds were purchased from ChemDiv (San Diego, CA). The catechol estrogen, 2-hydroxyestradiol (2-CE) was purchased from Steraloids, Inc. (Rhode Island, USA).

### Parasite Culture and Microscopy

The parasite strains NF54 and NF54 transfected with pHLIDH were grown in 5% hematocrit in RPMI 1640 (Invitrogen/Life Technologies) supplemented with 0.5% Albumax II (Invitrogen/Life Technologies), 0.25% sodium bicarbonate (standard media), and 0.01 mg/ml gentamycin. Human red blood cells for culture were obtained from human volunteers, cleared of leukocytes by passage through a Sepacell R-500 column (Baxter Health Care), and washed three times in RPMI 1640. Parasites were grown in sealed culture flasks under an atmosphere of 90% nitrogen, 5% oxygen, and 5% carbon dioxide. Parasitemias were maintained between 1 and 10%. Fixed parasites were stained with Giemsa to allow microscopic analysis of cultures using an Olympus BX40 compound microscope.

### *P. falciparum* ACβ Cloning

For cloning of *Pf*ACβ, we used the Gateway System (Invitrogen). A region encoding the N-terminal catalytic domain (AA 1–785) of gene PF3D7_0802600 (MAL8P1.150) was amplified with the following primer pair:

*P. falciparum* ACβ*:* FWD caccATGCTGAAAAATATCTTCTCCGAGTACC REV ttaGCCGATCGGGGAGTAAATTTTGATCAG.

A synthetic gene with mammalian codon usage was used as the template. A 4-nucleotide addition was included in the FWD primer for directional topoisomerase-based cloning, and a stop codon was included in the REV primer. Following the PCR reaction, fragments were resolved on a 1% Agarose gel. Bands corresponding to the appropriate size were excised and fragments were gel-purified (Qiagen gel purification kit). After quantification by gel electrophoresis and comparison to a High Mass Ladder (Invitrogen), 10 ng of each fragment was used in a 2-hr topoisomerase-based cloning reaction with pENTR/TEV-D-TOPO (Invitrogen). Two microliters of the cloning reaction was transformed into TOP10 *E. coli* (Invitrogen). Colonies were screened by restriction digest, and positive clones were sequenced using M13 forward and M13 reverse primers and multiple gene-specific primers. Clones found to be correct by sequencing were subsequently recombined into the “destination” vector pDEST20 (N-terminal GST tag) using a 1-hr LR Clonase II recombination reaction (Invitrogen).

pDEST20-*Pf*ACβ plasmid was transformed into DH10Bac *E. coli* (Invitrogen). Transformed bacteria were plated onto LB agar plates containing 50 µg/mL kanamycin (Sigma-Aldrich), 7 µg/mL gentamicin (Sigma-Aldrich), 10 µg/mL tetracycline (Sigma-Aldrich), 100 µg/mL Bluo-gal (Invitrogen), and 40 µg/mL isopropyl-β-D-1-thiogalactopyranoside (Sigma-Aldrich). White colonies, indicative of successful bacmid recombination, were picked and streaked on fresh plates to confirm the phenotype. Blue colonies were streaked on a separate area of the same plate as a control. Confirmed white colonies were cultured in 500 mL of LB containing 50 µg/mL kanamycin, 7 µg/mL gentamicin, and 10 µg/mL tetracycline.

Subsequently, bacmid DNA was isolated from the cell pellet using the NucleoBond Bac 100 DNA isolation kit (Macherey-Nagel). Isolated bacmid DNA was immediately transfected into *Sf*9 cells plated at ∼80% confluency on a 6-well plate (Becton-Dickenson) using Cellfectin reagent (Invitrogen). After transfection, successful recombination of bacmid DNA was confirmed by PCR analysis using M13 forward (Invitrogen), M13 reverse (Invitrogen), and the *Pf*ACβ FWD primer indicated above. Four days post-transfection, cells showed significant signs of baculovirus infection. Cell media containing recombinant baculovirus was harvested and clarified by centrifugation at ∼1,000×g. This P1 baculovirus stock was amplified first in a volume of 20 mL (400 µL P1 baculovirus was added) and subsequently in a volume of 500 mL (10 mL P2 baculovirus was added). For expression studies, 25 mL P3 baculovirus was added per liter of insect cells (either *Sf9* or Hi-Five).

### Heterologous Protein Expression

Insect cells are a proven system for expression and characterization of adenylyl cyclases [42]. Hi-Five cells at a density of 1×10^6^ cells/mL were infected with GST-*Pf*ACβ_1-785_ baculovirus at a concentration of 25 mL P3 baculovirus/L of culture. Infected cells were cultured for 40 hrs and harvested by centrifugation at ∼1000×g. Cells were frozen in liquid nitrogen and stored. Frozen pellets were resuspended in lysis buffer containing 50 mM Tris (pH 7.5), 5 mM DTT, 2 M NaCl, 10 µg/mL aprotinin/leupeptin, 1 mM PMSF, 1 mM benzamidine, 10 mM β-mercaptoethanol at a ratio of ∼10 mL lysis buffer/100 mL of pelleted culture. This lysate was sonicated 5 times at 10-second intervals at 12 watts with a Misonix Microson cell disruptor. The sonicated lysate was clarified by centrifugation at 100,000×g using a Ti-75 rotor (Beckman). The resulting supernatant was passed over a Superdex G-25 column with a 5-mL bed volume for further clarification. The clarified lysate was incubated on ice with minor agitation for 1 hr with 1 mL (packed volume) of glutathione sepharose 4B (Amersham) per 100 mL of lysate. The lysate was allowed to flow through, and the resin was washed with 3×10 bed volumes of lysis buffer. Finally, bound protein was eluted with 15 mM reduced glutathione in lysis buffer in 1 bed volume fractions. *Pf*ACβ protein was detected by activity and anti-GST Western blot (data not shown). *Pf*ACα pressed as previously described [22].

### Radioactivity-based Two-Column Adenylyl Cyclase Assay

Adenylyl cyclase assays with purified *Pf*ACβ and *Pf*ACα were performed according to the method of Salomon [43]. Purified GST-*Pf*ACβ. 50-500 ng) was incubated in 50 mM Tris, pH 7.5 (unless otherwise indicated), 1 mM DTT, 300 mM NaCl, 10 mM MnCl_2_ and 2.5 mM ATP (unless otherwise indicated) with ∼1,000,000 cpm [α-^32^P]ATP (Perkin Elmer) and ∼5,000 cpm [^3^H]cAMP (Perkin Elmer). (Tris buffers were pH-adjusted at room temperature for use at 37°C.) Reactions were performed in 100 µL for 20 minutes at 37°C and stopped with 150 µL 1.5% SDS. Product [^32^P]cAMP was separated from substrate [α-^32^P]ATP by sequential column chromatography over dowex 50WX4-400 resin (Fluka) followed by aluminum oxide resin (Sigma). Product [^32^P]cAMP was eluted from dowex, directly onto the alumina by water, and the cAMP was eluted from alumina by 0.1 M imidazole, pH = 7.3.

### Viability Assays

The NF54 strain transfected with pHLIDH expresses the firefly luciferase gene under the control of the constitutively active Hrp3 promoter [44]. This strain of parasites was created by transfection and stable integration of the plasmid pHLIDH into the genome of the NF54 wildtype parasite line. pHLIDH is a derivative of the pHLH-1 plasmid [44], in which the drug selectable marker *hdhfr* was inserted under the control of the PcDT5′ promoter [45]. Parasites were plated on day 0 at 1% parasitemia in 96-well plates in standard media in the presence of the indicated concentrations of DMSO (vehicle control), KH7, 2-CE, or KH7.15. Media plus compounds were replenished on day 1. On day 2, red blood cells were lysed with Bright-Glo Lysis Buffer (Promega), and luminescence was read using a luminometer (Molecular Devices) after injection with 10 µl Bright-Glo Luciferase Reagent (Promega) for a 2-sec integration time and a 15-sec read time. Data shown are normalized to the luminescence of vehicle-treated control parasites.

### Parasite Synchronization

NF54 parasites were synchronized as described [46]. Briefly, parasites in cultured RBCs were centrifuged for 4 min at 4000 rpm. The pellet was layered atop a 40%/70% Percoll-Sorbitol gradient and centrifuged for 20 min at 10,000 rpm. The late-stage fraction at the interface of the gradient was collected, washed in media, and reconstituted with fresh RBCs and media. Following erythrocyte invasion, the synchronized culture was expanded into 6 20-mL cultures at 3% parasitemia. At each indicated time point, one 20-ml culture was centrifuged for 2 min at 4000 rpm. The pellet was resuspended in 500 µl phosphate-buffered saline (PBS), and RBCs were lysed with 10 µl 10% saponin and microcentrifuged for 2 min at 13,000 rpm. The supernatant was aspirated, and pellets were frozen at −80°C until all time points were collected.

### Protection of Human Subjects

Blood was purchased from the New York City Blood Center or obtained from healthy human volunteers for use in parasite culture. A protocol for acquisition and use of human blood has been approved and is on file with the Internal Review board at Weill Medical College of Cornell University (Protocol #0010004662). For blood purchased from the New York City Blood Center (NYBC), contact of blood donors will not be attempted and is not necessary for the livelihood of the study. Informed consent is not required (other than NYBC in-house protocol). The blood will be used for research purposes only - solely for *in vitro* culture of *Plasmodium falciparum –* and not for transfusion into humans or animals. NYBC policy states that only surplus blood will be made available for research purposes, and thus this study will not compromise blood supplies. Blood will be used for research purposes only - solely for *in vitro* culture of *Plasmodium falciparum* - not for transfusion into humans or animals. The blood purchased from NYBC will only be used as a resource for propagation of malaria parasites and no data will be collected with regard to the blood itself. Therefore the inclusion of women, minorities or children is not applicable.

### Ethics Statement

Blood used in parasite cultures was obtained under a protocol approved by and on file with the Internal Review board at Weill Medical College of Cornell University or at New York Blood Center. All donors gave prior written consent.

## Supporting Information

Figure S1 **Structures of compounds used in this study.** 2-Catechol Estrogen (A), KH7 (B), KH7.15 (C), KH7.01 (D), KH7.02 (E), KH7.03 (F), KH7.04 (G), KH7.05 (H), KH7.08 (I), KH7.09 (J).
(TIF)

Figure S2 **Comparison of luciferase-based viability assay with tritiated hypoxanthine uptake-based assay.** Parasite viability with measured with the luciferase-based (yellow curves) or tritiated hypoxanthine-based viability assay (red curves) in the presence of increasing concentrations of chloroquine (A), quinine (B), mefloquine (C), and artemisinin (D). Best-fit curves are shown. Y-axis is percentage assay readout; X-axis is log_10_ drug concentration. EC_50_s for each drug are shown below the figure. Best-fit curves are highly similar for each drug.
(TIFF)

Figure S3 **Expression levels of PfACβ in the red blood cell.** RT-PCR using PfACβ-specific primers confirms publicly available microarray data [23], [24]. Both primer sets 1 (blue bars) and 2 (red bars) amplify high levels of PfACβ mRNA in the late trophozoite and schizont stages of the parasite. Representative photos of Giemsa-stained parasites corresponding to the time of RNA extraction for the RT-PCR analysis are shown below the graph.
(TIFF)

Figure S4 **The solubility of His-tagged** ***Pf*** **ACβ_1-785_ is increased by high salt conditions.** (Similar results were obtained with GST-*Pf*ACβ_1-785_). Hi-5 insect cells were infected with His-tagged *Pf*ACβ_1-785_ baculovirus and harvested after 42 hrs (determined to be the optimal time for maximal activity and expression of intact protein). Cell pellets were resuspended in a lysis buffer containing 50 mM Tris (pH = 7.5), 10 µg/mL aprotinin/leupetin, 1 mM PMSF, 1 mM benzamidine, 200 mM NaCl, and 1 mM DTT at ∼10 mL lysis buffer/100 mL of pelleted culture. This lysate was sonicated five times at 10-second intervals at 12 watts with a Misonix Microson cell disruptor. Sonicated lysate was clarified by centrifugation at 100,000×g using a Ti-75 rotor (Beckman). The pellet fraction was resuspended in lysis buffer and adenylyl cyclase activity corresponding to *Pf*ACβ_1-785_ activity remained in the insoluble pellet fraction. The various additives indicated above were added to the resuspended pellet fraction, and the solution was again clarified by centrifugation. Soluble fractions were assayed for adenylyl cyclase activity. This was used as a measure of *Pf*ACβ_1-785_ amount. Only 2 M NaCl significantly solublized *Pf*ACβ_1-785_.
(TIFF)

Figure S5 **Effect of KH7-like compounds on parasite viability.** *P. falciparum* cultures were maintained in a 96-well plate in the presence of 40 µM of the indicated compound. Luminescence was measured after 48 hrs. Reactions were performed in duplicate.
(TIF)
