# Supplementary figures and images for: Characterization of Plasmodium falciparum Adenylyl Cyclase-β and Its Role in Erythrocytic Stage Parasites
Source: PLoS One. 2012 Jun 26;7(6):e39769. doi: 10.1371/journal.pone.0039769 (PMC3383692; doi:10.1371/journal.pone.0039769)

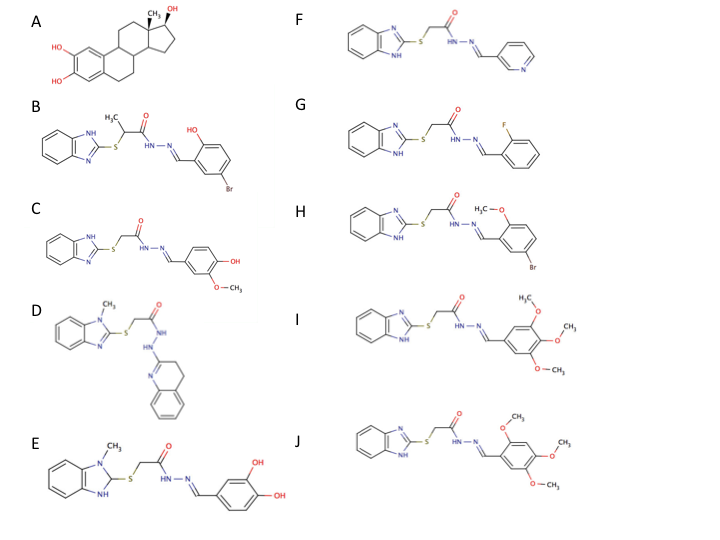

Supplement: Figure S1 — Structures of compounds used in this study. 2-Catechol Estrogen (A), KH7 (B), KH7.15 (C), KH7.01 (D), KH7.02 (E), KH7.03 (F), KH7.04 (G), KH7.05 (H), KH7.08 (I), KH7.09 (J). (TIF) [file pone.0039769.s001.tif]

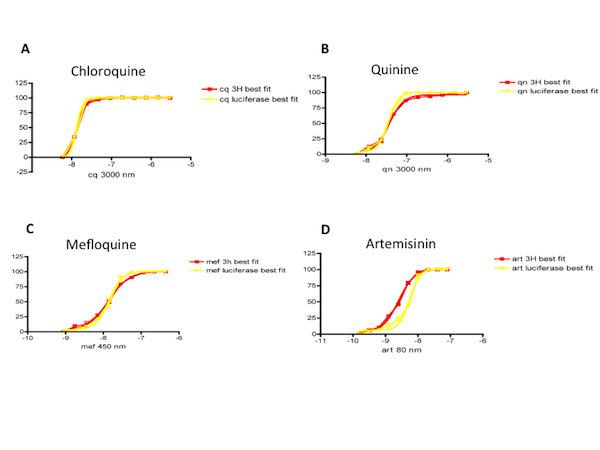

Supplement: Figure S2 — Comparison of luciferase-based viability assay with tritiated hypoxanthine uptake-based assay. Parasite viability with measured with the luciferase-based (yellow curves) or tritiated hypoxanthine-based viability assay (red curves) in the presence of increasing concentrations of chloroquine (A), quinine (B), mefloquine (C), and artemisinin (D). Best-fit curves are shown. Y-axis is percentage assay readout; X-axis is log10 drug concentration. EC50s for each drug are shown below the figure. Best-fit curves are highly similar for each drug. (TIFF) [file pone.0039769.s002.tiff]

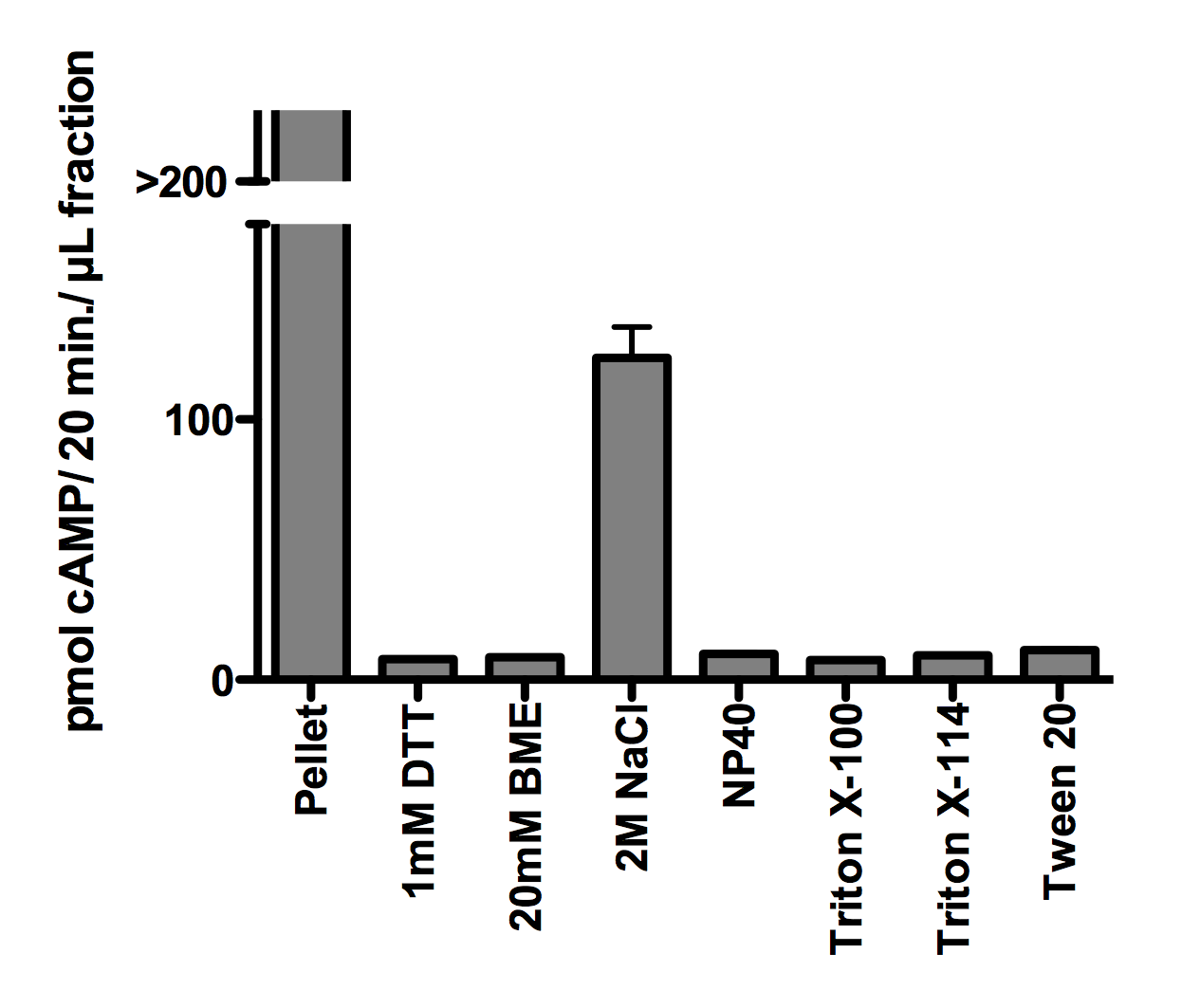

Supplement: Figure S3 — Expression levels of PfACβ in the red blood cell. RT-PCR using PfACβ-specific primers confirms publicly available microarray data [23], [24]. Both primer sets 1 (blue bars) and 2 (red bars) amplify high levels of PfACβ mRNA in the late trophozoite and schizont stages of the parasite. Representative photos of Giemsa-stained parasites corresponding to the time of RNA extraction for the RT-PCR analysis are shown below the graph. (TIFF) [file pone.0039769.s003.tiff]

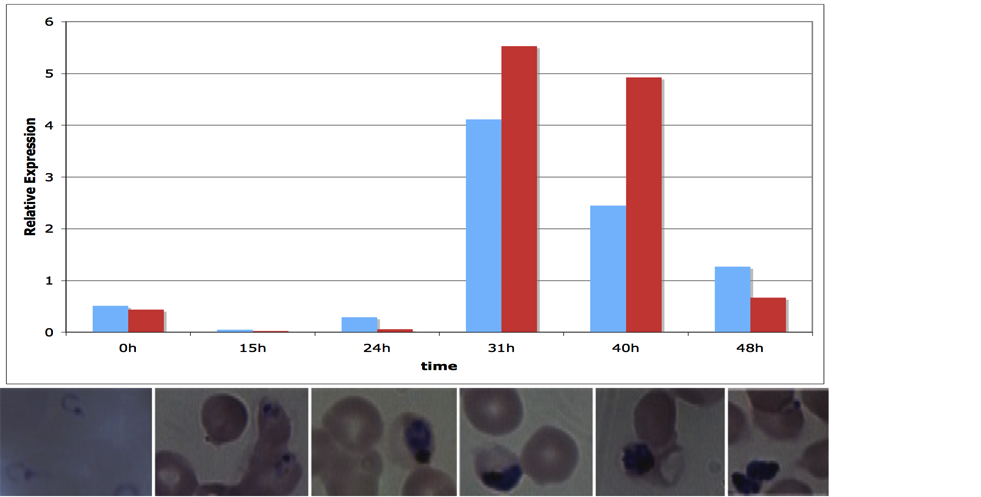

Supplement: Figure S4 — The solubility of His-tagged Pf ACβ1-785 is increased by high salt conditions. (Similar results were obtained with GST-PfACβ1-785). Hi-5 insect cells were infected with His-tagged PfACβ1-785 baculovirus and harvested after 42 hrs (determined to be the optimal time for maximal activity and expression of intact protein). Cell pellets were resuspended in a lysis buffer containing 50 mM Tris (pH = 7.5), 10 µg/mL aprotinin/leupetin, 1 mM PMSF, 1 mM benzamidine, 200 mM NaCl, and 1 mM DTT at ∼10 mL lysis buffer/100 mL of pelleted culture. This lysate was sonicated five times at 10-second intervals at 12 watts with a Misonix Microson cell disruptor. Sonicated lysate was clarified by centrifugation at 100,000×g using a Ti-75 rotor (Beckman). The pellet fraction was resuspended in lysis buffer and adenylyl cyclase activity corresponding to PfACβ1-785 activity remained in the insoluble pellet fraction. The various additives indicated above were added to the resuspended pellet fraction, and the solution was again clarified by centrifugation. Soluble fractions were assayed for adenylyl cyclase activity. This was used as a measure of PfACβ1-785 amount. Only 2 M NaCl significantly solublized PfACβ1-785. (TIFF) [file pone.0039769.s004.tiff]

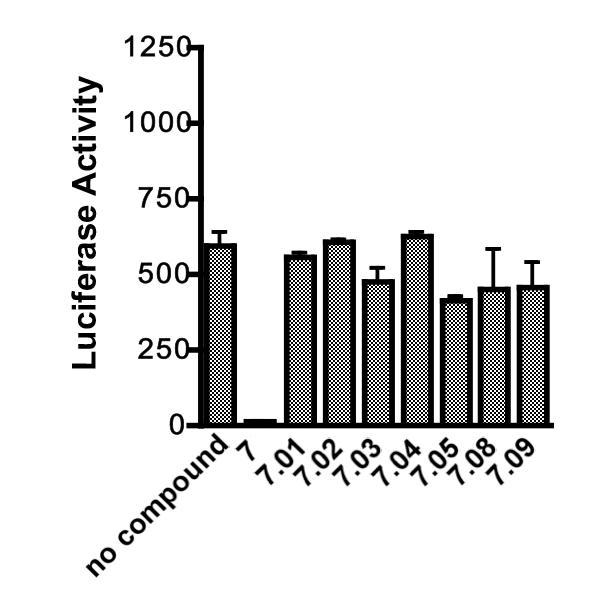

Supplement: Figure S5 — Effect of KH7-like compounds on parasite viability. P. falciparum cultures were maintained in a 96-well plate in the presence of 40 µM of the indicated compound. Luminescence was measured after 48 hrs. Reactions were performed in duplicate. (TIF) [file pone.0039769.s005.tif]
